# Association between a mobile team intervention in Swedish municipal home care and the effect on emergency department visits and hospitalizations among older adults

**DOI:** 10.1186/s12913-025-12843-1

**Published:** 2025-05-10

**Authors:** Karin Erwander, Kjell Ivarsson, Björn Agvall

**Affiliations:** 1 Department of Clinical Sciences, Lund University, Lund, Sweden; 2 Department of Research and Development, Halmstad, Region Halland Sweden; 3 Department of Clinical Sciences, Center for Primary Health Care Research, Malmö, Lund University, Malmö, Sweden

**Keywords:** Elderly, Home health care, Mobile teams, Emergency care, ED visits

## Abstract

**Background:**

Elderly individuals with chronic conditions or acute illnesses are major drivers of hospitalization, with frail patients frequently utilizing emergency department (ED) services. To ease this burden, many countries offer home-based medical services. In Region Halland, Sweden, a mobile team intervention in municipal home care (MHC) was introduced to support frail elderly patients. This study aimed to assess whether the intervention reduced ED visits and hospitalizations among MHC recipients.

**Methods:**

The study population consisted of all patients aged ≥ 65 years enrolled in MHC in Halmstad, Sweden, from October 2014 - April 2016. Healthcare utilization during the seven months prior to the initiation of the intervention (October 2014 - April 2015) constituted the pre-intervention group and were compared with healthcare consumption during a seasonally matched seven-month period after the launch of the intervention (October 2015-April 2016). The primary outcome was the number of adverse events, defined as unplanned ED visits or hospital admission. Negative binomial regression was used to assess the association between exposure and adverse events, presented as Incidence Rate Ratios (IRRs) with 95% confidence intervals (CIs).

**Results:**

A total of 2163 patients were included in the pre-intervention group, and 2197 patients in the intervention group. Both groups had a mean age of 84 years, with no significant differences regarding sex. In the pre-intervention group, 64% had severe comorbidities, compared to 66% in the intervention group. Primary care home visits by physicians increased from an average of 0.9 in the pre-intervention group to 1.1 in the intervention group (*p* < 0.001). Risk for adverse events was elevated among patients with severe comorbidities (IRR = 3.14, 95% CI: 1.91–5.15, *p* = < 0.001). There was a slight decrease in the incidence rate for the intervention group; however, this reduction was not statistically significant (IRR = 0.91, 95% CI: 0.82–1.01, *p* = 0.09).

**Conclusion:**

The mobile team intervention in MHC did not significantly reduce ED visits or hospitalizations among elderly MHC recipients, suggesting that physician-led interventions alone may be insufficient to lower acute care utilization in this population. This highlights the complexity of care needs among frail older adults and suggests that a more comprehensive, multidisciplinary approach may be required to achieve meaningful reductions in emergency care use.

## Keypoints

• The intervention with a mobile team in municipality home care had limited impact on ED visits and hospitalization.

• The mobile team intervention was associated with increased encounters with primary care physicians.

• Patients with severe comorbidities exhibited the highest risk for adverse events.

## Background

The aging population and the increasing use of the emergency departments (ED) and hospitalization present a major challenge for the healthcare system [1, 2]. Healthcare utilization rises with age, with elderly patients accounting for approximately 20% of all ED visits [3–5]. Elderly individuals who experience deterioration in their chronic conditions or develop acute illnesses are the primary drivers of hospitalization [6]. ED utilization is particularly high among frail elderly patients with multiple comorbidities and is further exacerbated by a lack of effective care planning [7–9]. Frequent ED visits and admissions place a significant strain on the healthcare system, prompting numerous initiatives to reduce such occurrences.

Previous studies have identified three major intervention categories aimed at reducing ED visits: case management, individualized care plans and information sharing [10, 11]. In recent years, mobile teams and hospital-at-home have been proposed as alternative strategies to prevent unnecessary ED visits and hospital admissions [12]. The literature on mobile teams and their impact on ED visits is mixed. A study has demonstrated positive effects such as the early detection of patient deterioration and improved care coordination, particularly among high-risk older adults [13]. Another study have reported that while mobile teams may improve outcomes such as follow-up care or patient satisfaction, their overall effect on reducing ED visits is not always statistically significant [14]. Additionally, research focusing on cost and resource considerations indicates that mobile teams can be resource intensive, and their cost-effectiveness may be limited if they are not well targeted, especially among populations that are not the highest utilizers of ED services [15].

Consequently, many countries now offer home-based medical services with the intention to prevent avoidable ED visits and hospital admissions, as the hospital-at-home model can benefit patient well-being and reduce the need for hospitalization [16, 17]. In Region Halland, Sweden, a mobile team intervention for elderly patients receiving municipal home care (MHC) was introduced in May 2015. The aim was to support MHC by increasing resources and facilitating greater physician involvement in the care of elderly and frail patients. The aim of this study was to assess whether a mobile team intervention led to positive outcomes by reducing ED visits and hospitalizations among older adults receiving MHC.

## Methods

### Setting

Region Halland, located in the southwest of Sweden, is home to just over 330 000 residents. Halmstad the largest municipality in the region, had approximately 100 000 inhabitants in 2016. The region houses three hospitals, with Halmstad Hospital being the largest, featuring 18 inpatient units and 335 beds.

### Data source

Region Halland utilizes pseudonymized data from the Regional Healthcare Information Platform (RHIP), which encompasses clinical, operational, and financial information of individuals receiving treatment since 2011 across all healthcare facilities within the region. RHIP serves as the primary data source for this study and includes information from primary care, emergency departments, hospital admissions, outpatient care, and inpatient care – providing a comprehensive view of all patient encounters, resource allocation, diagnosis, and capacity. The registry for patients receiving municipal home health care was extracted from electronical medical records of Halmstad municipality.

### Study population

The study population consisted of all the patients aged ≥ 65 years who were registered in MHC in Halmstad from October 2014 - April 2016. Healthcare utilization patterns during the seven months before the implementation of the mobile team intervention (October 2014 - April 2015) were compared with those during a seasonally matched seven-month period after the launch of the intervention (October 2015-April 2016). This seasonal matching helped account for potential variations in healthcare utilization due to seasonal factors. All included patients were residents of Halmstad municipality and received their healthcare within the municipality. No patients were excluded.

### Study process

The data collection was generated from the RHIP. The anonymized data was collected on 10th of May 2023 and the authors had no access to information that could identify individual participants during or after data collection. Variables extracted were age, gender, ED visits, admission, bed-days, contacts with out-patient care and primary care during the study period. Comorbidities were calculated using CCI [18]. According to the WHO classification patients *≥* 65 years of age were classified as older adults and then categorized into three different age groups: 65–74 years, 75–84 years and > 85 years [19]. The number of patients hospitalized during the period was collected, and the number of hospital bed-days per patient was recorded. The number of ED visits, hospital out-patient visits and primary care visits was documented and categorized into visits to primary care physicians (PCP) and visits to primary care nurses.

High utilizers were identified in the municipality of Halmstad during 2016, and defined as patients who had five or more visits to their primary care physician, four or more ED visits, three or more hospital admissions during each of the study periods [20, 21].

### Regular healthcare in the pre-intervention group

The pre-intervention group included patients discharged from in-patient care to their homes or those requiring follow-up from MHC. It comprised patients living at home who experienced health deterioration leading to a need for MHC, with referrals originating from primary care. Routine healthcare in this group consisted primarily of medical follow-up in primary care, though in some cases, parallel follow-up in hospital outpatient settings was provided. Standard MHC involved managing various chronic conditions in older adults, such as treating leg ulcers, taking blood tests, managing medications, monitoring and regulating blood pressure, addressing heart failure and pulmonary diseases, and ensuring adherence to established care plans. The nurses were authorized to collect blood samples following orders from primary care or hospital physicians. Due to routine healthcare procedures, municipal nurses often encountered challenges in reaching a physician, relying on fax communication and lacking direct phone contact when addressing patient care issues.

### Description of the mobile team intervention

The mobile team intervention in Halmstad was fully launched in May 2015 to support nurses in MHC. The goal was to enable patients to receive care at home with the assistance of MHC nurses working in collaboration with physicians.

The intervention included all patients with access to MHC in Halmstad, excluding those who lived in or moved to nursing homes during the study period. The primary responsibilities of the physicians in the mobile team were to develop care and treatment plans for patients in need of MHC, provide consultation and conduct home visits on short notice. The goal of these treatment plans was to ensure that if a patient’s condition deteriorated at home, there was already a predefined plan to utilize available MHC resources, thereby avoiding ED visits and hospitalization. Prompt physician consultations on short notice aimed to enhance patient safety and comfort.

The intervention program had a physician on duty during office hours (0900–1600, Monday through Friday). Outside of these hours, the physician in the mobile team was not available for consultations; instead, the on-call physician within the municipality handled patient issues based on the existing care plans. Three specialized physicians were employed in the mobile team intervention– though not all were general practitioners, as some had other specialties. When nurses encountered an issue, they could not resolve independently, they contacted the mobile team physician for a phone consultation. For patient deteriorations occurring outside office hours, the MHC nurse would contact the municipal on-call physician, who could then make short-term decisions to safeguard the patient’s health until the intervention team was available again.

The intervention provided patients with easy telephone access to MHC nurses and allowed for the nurses to conduct physical examinations within the same day, including the assessment of vital parameters and blood sampling, but no bedside blood tests. The intervention was designed to facilitate timely communication and information sharing, and MHC nurses could initiate actions based on pre-established treatment plans, while physicians were available for consultations on short notice.

### Statistical analysis

Descriptive statistics were used to characterize patient demographics. Continuous variables were described as means *±* standard deviation (SD) and analyzed using Student’s t-test. Categorical variables were summarized using frequency and percentages and compared using Pearsons’s Chi-Square tests. To compare the proportion of patients experiencing *> 1 adverse event between the pre-intervention and intervention group*,* a separate chi-square test was conducted as a supplementary group-level analysis.*

Comorbidities were calculated based on all primary and secondary diagnoses across visits to all caregivers and categorized as mild (CCI score 1–2), moderate (CCI score 3–4) and severe (CCI score ≥ 5) according to CCI [18]. Overall care utilization was examined during both the pre-intervention and post-intervention periods of the mobile team intervention.

The primary outcome was the number of adverse events, defined as unplanned ED visits or hospital admission. Secondary outcomes included demographic characteristics and overall healthcare utilization, such as bed-days, out-patient visits and visits to primary care.

To examine the association between the intervention and adverse events, a negative binomial regression analysis was conducted to adjusting for age, sex and comorbidities. Negative binomial regression was selected over Poisson regression due to overdispersion in the data, where the variance exceeded the mean.

A *p*-value < 0.05 was considered statistically significant. The analyses were executed with IBM SPSS Statistics 28, Armonk, New York, USA. There were no missing values in the data collection. The Ethical Review Agency in Sweden granted ethical approval for the study (reference number 2016/20).

## Results

In the pre-intervention group, 2163 patients were included, while the intervention group comprised 2197 patients who had access to the mobile team. The mean age was 84 years in both groups, and their baseline characteristics are displayed in Table 1. In the pre-intervention group, 64% of participants were classified as having severe comorbidities according to CCI, compared to 66% in the intervention group. No statistically significant differences were observed between the two groups regarding gender, age or comorbidity distribution. The proportion of high utilizers was 4% in both groups regarding frequent visits to the ED, 21% (473) of the patients in the intervention group made *≥* 5 visits to PCP vs. 18% (395) in the pre-intervention group. For both the pre-intervention group and the intervention group 8% of the patients had *≥* 3 admissions to the hospital during the study period.

**Table 1:** Baseline characteristics for the pre-intervention group and the intervention group

	Pre-intervention group	Intervention group	Total	*p*-value
*Total*,* n (%)*	2163 (50)	2197 (50)	4360	
*Gender*				
Female	1406 (65)	1402 (64)	2808 (64)	0.41
*Age*,* mean (SD)*	84.5 (7.7)	84.1 (7.8)	84.3 (7.7)	0.22
65–74, n (%)	277 (13)	302 (14)	579 (13)	0.36
75–84, n (%)	701 (32)	739 (33)	1440 (33)	0.39
85-, n (%)	1185 (55)	1156 (53)	2341 (54)	0.15
*Charlson Comorbidity Index*				
Mild, n (%)	24 (1)	26 (1)	50 (1)	0.82
Moderate, n (%)	746 (35)	719 (33)	1465 (34)	0.22
Severe, n (%)	1393 (64)	1452 (66)	2845 (65)	0.24
*High utilizers*				
* ≥* 4 ED visits, n (%)	90 (0.04)	97 (0.04)	187 (0.04)	0.68
* ≥* 3 IP-visits, n (%)	175 (0.08)	185 (0.08)	360 (0.08)	0.69
* ≥* 5 PC-visits, n (%)	395 (0.18)	473 (0.21)	868 (0.2)	0.007

*ED* Emergency Department, *SD* Standard Deviation, *CCI* Charlson Comorbidity Index, *IP* In patient (admission), *PCP* Primary Care Physician

Overall care consumption between the intervention group and the pre-intervention group is presented in Table 2. There was a statistically significant increase in PCP home visits in the intervention group compared to the pre-intervention group, as well as a decrease in nurse visits in primary care. No statistically significant difference was found between the groups regarding ED visits, hospital admissions or bed-days.

**Table 2:** Illustrate care consumption for the pre-intervention group and the intervention group during the study period

Care consumption	Pre-intervention group (*n* = 2163)			Intervention group (*n* = 2197)			Total			*p*-value
	n	Mean	SD	n	Mean	SD	n	Mean	SD	
*In-patient care*										
ED visits	1713	0.8	1.3	1776	0.8	1.4	3498	0.8	1.3	0.37
Admissions	1610	0.7	1.3	1594	0.7	1.2	3204	0.7	1.2	0.84
Bed-days	11 093	5.1	10.8	11 026	5.0	10.0	22 119	5.1	10.4	0.65
*Out-patient care*										
Physician visits	2083	1.0	2.1	2498	1.1	2.1	4581	1.0	2.1	< 0.001
*Primary care*										
Nurse visits	7466	3.4	12.0	4940	2.2	5.5	12 406	2.8	9.3	< 0.001
PCP visits	5570	2.6	3.2	6173	2.8	3.3	11 743	2.7	3.2	0.06
PCP home-visits	1950	0.9	2.3	2514	1.1	2.4	4464	1.0	2.4	< 0.001

*ED* Emergency Department, *PCP* primary care physician, *SD* Standard Deviation

A comparison of adverse event frequency, defined as *> 1 unplanned ED visit of hospital admission is presented in* Table 3. *The proportion of patients experiencing at least one adverse event was similar in both groups: 46*,*7% in the pre-intervention group and 45*,*9% in the intervention group (p = 0.57)*,* indicating no statistically significant difference.*

**Table 3:** Proportion of patients with ≥ 1 adverse event in pre- and intervention groups

Group	Total patients, *n*	Patients with *≥* 1 adverse event, n	% with adverse event*	*p*-value**
Pre-intervention group	2163	1011	46.7	
Intervention group	2197	1008	45.9	
Total study group	4360	2019	46.3	0.57

* Adverse events are defined as ED visits or IP-visits

** *p*-value from Pearson Chi-Square test comparing proportion of patients with ≥ 1 adverse event between groups

Risk for adverse events was further analysed using negative binomial regression. As shown in Table 4, patients with severe comorbidities had a statistically significant higher risk for adverse events (Incidence rate ratio (IRR) = 3.14, 95% CI: 1.91–5.15, *p* = < 0.001). There was a slight, non-significant reduction in the incidence rate for the intervention group (IRR = 0.91, 95% CI: 0.82–1.01, *p* = 0.09).

**Table 4:** Negative binomial regression analysis of risk for adverse events* and the association between age, sex, comorbidities and the intervention group

Predictor	IRR	95% C.I.	*p*-value
*Study period*			
Pre-intervention group	Ref.		0.09
Intervention group	0.91	0.82–1.01	
*Age (years)*	0.95	0.95–0.96	< 0.001
*Sex*			
Male	Ref.		< 0.001
Female	0.68	0.59–0.77	
*CCI*			
Mild	Ref.		< 0.001
Moderate	2.32	1.41–3.81	
Severe	3.14	1.91–5.15	

*IRR* Incidence Rate Ratio, *CCI* Charlson Comorbidity Index

*Adverse events are defined as ED visits or IP-visits

## Discussion

This study evaluated the associations between a mobile team intervention in MHC in Region Halland, Sweden, on reducing ED visits and hospitalizations among elderly patients. Both the pre-intervention group and the intervention group had a mean age of 84 years and similar comorbidities. Primary care home visits by physicians increased significantly in the intervention period. While patients with severe comorbidities had a higher risk of adverse events, no statistically significant reduction in ED visits or hospital admissions was observed in the intervention period. The findings suggest that physician-led interventions alone may not be sufficient to reduce acute care utilization.

The absence of significant reduction in ED visits and hospitalization suggest that the mobile team intervention did not achieve its primary objective of reducing acute care utilization among elderly MHC recipients. The significant increase in PCP home visits indicates improved access to primary care, however, this enhanced access may not have been sufficient to avert acute episodes leading to ED visits or hospitalizations. The elevated risk of adverse events among patients with severe comorbidities reinforces the complexity of managing this vulnerable population. The slight decrease in adverse event incidence in the intervention group, while not statistically significant, suggest a potential trend that might become more apparent in a larger study. These findings align with previous research, indicating that outpatient interventions often have minimal impact on reducing ED visits unless care plans are highly individualized [15].

In Sweden and several other countries, mobile teams, hospital-at-home and similar programs have been implemented with the expectation of reducing acute care utilization. Several studies have shown the ability of home healthcare to successfully provide quality care in the outpatient setting, thereby decreasing unnecessary utilization of inpatient resources and generating an overall cost savings for the healthcare system [22–24]. For example, in Halmstad, Region Halland, it was estimated that the mobile team intervention in MHC could save three hospital beds and €300 000 annually – a key rationale behind its political adaptation. Yet our results indicate no significant difference in inpatient hospitalizations or ED visits between the groups. As many countries struggle with the rising costs of healthcare, expanding of home healthcare services have been proposed as a potential method to deliver care in a more cost-effective manner. Several studies have shown the importance of mobile teams and that the collaborations between physicians and nurses in MHC play a crucial role for the ability to successfully provide quality care in the outpatient setting [25, 26].

Several factors might explain these results. First the mobile team intervention may have improved care coordination and primary care accessibility without directly impacting the underlying clinical trajectories that lead to acute exacerbations. Second the intervention might have been more effective if targeted more narrowly at patients with the highest risk, for example high-utilizers, given that a high proportion of the cohort already had severe comorbidities. Third, operational factors such as the limited availability of the intervention during off-hours may have diluted its potential impact on reducing acute care events. Despite the lack of significant reduction in acute care utilization, anecdotal reports from physicians and nurses suggest that the intervention has positively influenced patients’ quality of life. Although this study did not assess patient comfort or quality of life, previous research had highlighted these benefits, which should not be underestimated [27].

Overall, the challenges of an aging population and the frequent use of ED services persist. Future studies should focus on more targeted interventions for high-risk groups, include primary care physicians and consider operational enhancements to maximize the benefits of mobile team intervention in MHC. These efforts are crucial for developing cost-effective, high quality care pathways that address both clinical outcomes and patient well-being.

### Strengths and limitations

A key strength of this study is the use of a large, well-characterized cohort with comparable baseline characteristics, which enhances the validity of the findings. However, several limitations must be acknowledged.

First, the mobile team intervention in MHC was only available during weekdays and regular office hours, limiting its accessibility and reach. As a result, only a small portion of the target patient population could utilize the service, and relatively few contacts were recorded during the study period. Since the primary aim of the intervention was to reduce the need for ED visits and hospital admissions, this limited availability may have influenced the observed effects.

Second, the study lacked a matched comparison group from outside RH due to data availability constraints. This limits the generalizability of the findings and restricts the ability to fully evaluate the intervention’s impact relative to other populations or settings.

The retrospective design of the study, while inclusive of the entire eligible population reduce selection bias, still carries the risk of unmeasured confounding. The use of routinely collected administrative data may also have led to missing or incomplete information, potentially affecting the accuracy of some variables and outcomes.

Finally, an inherent limitation of the pre-post study design is the inability to account for secular trends, such as changes in healthcare delivery, system-level policies, or patient behavior over time, that may have influenced outcomes independently of the intervention. Therefore, all statistically significant findings should be interpreted as associations, and not as evidence of causality.

## Conclusion

The mobile team intervention did not significantly reduce adverse events, such as ED visits or hospitalizations. Patients with severe comorbidities had a significantly increased risk for adverse events. The increased engagement with primary care physicians suggests potential benefits in patient monitoring and ongoing care. Future healthcare delivery strategies should focus on more targeted interventions for high-risk subgroups and enhance collaboration with primary care. This approach could help determine whether modifications to the intervention might yield a more substantial reductions in acute care utilization.

## Data Availability

The datasets generated and/or analyzed during the current study are not publicly available due to the data being retrieved from patient’s hospital records which is included in the Swedish Health Care act which applies to Swedish secrecy act according to Swedish legislation. The data will be shared on reasonable request to the corresponding author. Not applicable.

## Abbreviations

CCI: Charlson Comorbidity Index
CI: Confidence interval
ED: Emergency Department
IP: In patient
IRR: Incidence Rate Ratio
PCP: Primary Care Physician
MHC: Municipality Home Care
RHIP: Regional Healthcare Information Platform
SD: Standard deviation
